# Epigenetic nature of *Arabidopsis thaliana* telomeres

**DOI:** 10.1093/plphys/kiac471

**Published:** 2022-10-11

**Authors:** María I Vaquero-Sedas, Miguel A Vega-Palas

**Affiliations:** Instituto de Bioquímica Vegetal y Fotosíntesis, CSIC-Universidad de Sevilla, IBVF (CSIC-US), Seville E41092, Spain; Instituto de Bioquímica Vegetal y Fotosíntesis, CSIC-Universidad de Sevilla, IBVF (CSIC-US), Seville E41092, Spain

## Abstract

The epigenetic features of defined chromosomal domains condition their biochemical and functional properties. Therefore, there is considerable interest in studying the epigenetic marks present at relevant chromosomal loci. Telomeric regions, which include telomeres and subtelomeres, have been traditionally considered heterochromatic. However, whereas the heterochromatic nature of subtelomeres has been widely accepted, the epigenetic status of telomeres remains controversial. Here, we studied the epigenetic features of Arabidopsis (*Arabidopsis thaliana*) telomeres by analyzing multiple genome-wide ChIP-seq experiments. Our analyses revealed that Arabidopsis telomeres are not significantly enriched either in euchromatic marks like H3K4me2, H3K9ac, and H3K27me3 or in heterochromatic marks such as H3K27me1 and H3K9me2. Thus, telomeric regions in Arabidopsis have a bimodal chromatin organization with telomeres lacking significant levels of canonical euchromatic and heterochromatic marks followed by heterochromatic subtelomeres. Since heterochromatin is known to influence telomere function, the heterochromatic modifications present at Arabidopsis subtelomeres could play a relevant role in telomere biology.

## Teaser

Telomeric regions in Arabidopsis have a bimodal chromatin with telomeres lacking substantial levels of canonical euchromatic and heterochromatic marks followed by heterochromatic subtelomeres.

## Introduction

Telomeres guarantee the replication of chromosome ends, prevent genome instability, and influence relevant biological processes like the proliferative capacity of stem cells, illness, aging, and cancer (Blackburn et al., 2015). In Arabidopsis, telomeres consist of tandem arrays of the plant type telomeric repeat unit (CCCTAAA/TTTAGGG) that spread 2.5–5 kb (Richards and Ausubel, 1988). These repeats are also present at internal chromosomal loci, where they have been related to genome instability. However, the function of these interstitial telomeric sequences (ITSs) remains largely unknown (Aksenova and Mirkin, 2019).

The length of telomeres and their chromatin organization influence telomere functions (Venditti et al., 1999; Nishibuchi and Déjardin, 2017; de Lange, 2018). The basic units of chromatin are the nucleosomes, which consist of 146 bp of DNA wrapped around a histone octamer containing two dimers of histones H2A–H2B and a tetramer of histones H3 and H4 (Luger et al., 1997). These basic chromatin units fold into more complex organizations that ultimately contribute to compact DNA and regulate its metabolism (Jung and Kim, 2021). Two main kinds of chromatin organizations are found within the nucleus of eukaryotic cells: euchromatin and heterochromatin. Euchromatin usually associates with single-copy sequences and has an open conformation that can allow transcription. In turn, heterochromatin associates with repetitive elements usually located at pericentromeres, is compact, and generally silenced. It can be observed in interphase nuclei as densely stained nuclear areas known as chromocenters (Nishibuchi and Déjardin, 2017; Vergara and Gutierrez, 2017). When euchromatin acquires a closed conformation by the action of the Polycomb Repressive Complexes 1 and 2 (PRC1 and PRC2), it is referred to as polycomb chromatin or facultative heterochromatin. This type of chromatin regulates development and differentiation by silencing specific genes through mechanisms that differ from those that operate at heterochromatin (Schuettengruber et al., 2017).

In Arabidopsis (*Arabidopsis thaliana*), euchromatin associates with different epigenetic marks that allow transcription including H3K4me1,2,3, H3K36me1,2,3, H4K20me2,3, and histones acetylation. When Arabidopsis euchromatin is labeled with the repressive H2AK121ub and H3K27me3 marks by the PRC1 and PRC2 complexes, respectively, it acquires a closed conformation that can lead to gene silencing. A closed conformation is also found within Arabidopsis heterochromatin, which silence mobile elements through the establishment of repressive marks such as DNA methylation, H3K9me2, and H3K27me1 (Fuchs et al., 2006; Roudier et al., 2011; Vergara and Gutierrez, 2017; Yin et al., 2021). Thus, the specific combinations of epigenetic marks at defined Arabidopsis domains condition their biochemical and functional properties. Therefore, there is interest in knowing the epigenetic characteristics of defined chromosomal loci.

One of the major Arabidopsis heterochromatic marks is cytosine methylation (Roudier et al., 2011; Vergara and Gutierrez, 2017). Arabidopsis has significant levels of cytosine methylation in the CG, CHG, and CHH contexts (where H is A, C, or T), which are established and/or maintained by specific DNA methyltransferases (Du et al., 2015). Methylation in all sequence contexts is established de novo by the DOMAINS REARRANGED METHYLTRANSFERASE 2 (DRM2) through the RNA-directed DNA methylation (RdDM) pathway. Then, methylation is spread and maintained by METHYLTRANSFERASE 1 (MET1), CHROMOMETHYLASES 2 and 3 (CMT2 and CMT3), and also DRM2. Whereas MET1 and CMT3 are the major Arabidopsis CG and CHG methyltransferases, respectively, CMT2 and DRM2 maintain methylation at CHH sites and, to a lower extent, at CHG sites. CMT3, CMT2, and DRM2 can associate with the heterochromatic H3K9me2 mark to maintain non-CGm (Law et al., 2011, 2013; Du et al., 2012; Stroud et al., 2014). In turn, the histone methyltransferases SU(VAR)3–9 HOMOLOG4/KRYPTONITE (SUVH4/KYP), SUVH5, and SUVH6 bind to methylated cytosines to maintain H3K9me2 (Jackson et al., 2002, 2004; Ebbs and Bender, 2006; Stroud et al., 2013). Thus, DNA and histone methyltransferases create a positive interdependent feedback loop that reinforces heterochromatin spreading and maintenance. Consequently, non-CGm is largely required for H3K9me2 and vice versa (Du et al., 2015; Wendte and Schmitz, 2018).

Arabidopsis heterochromatin is also characterized by H3K27me1, which is established by the ARABIDOPSIS TRITHORAX-RELATED PROTEINS 5 and 6 (ATXR5 and ATXR6). The establishment of H3K27me1 by these proteins has been shown to confer genome stability by inhibiting re-replication and transcription of mobile elements within heterochromatin. Whereas H3K27me1 is largely unaffected in *suvh4/5/6* and in DNA methyltransferases mutants, DNA methylation and H3K9me2 are largely unaffected in the *atxr5/6* mutant. However, DNA methyltransferases and SUVH4/5/6 are required for the over-replication of heterochromatin observed in *atxr5/6* (Mathieu et al., 2005; Jacob et al., 2009, 2010; Stroud et al., 2012a; Feng et al., 2017). Thus, although H3K27me1 and DNA methylation/H3K9me2 seem to be maintained by independent pathways, they contribute to silence mobile elements and to control DNA replication within heterochromatin.

The presence of the histone H3.1 variant also characterizes Arabidopsis heterochromatin. Whereas histone H3.1 associates with heterochromatic regions of the Arabidopsis genome, histone H3.3 is preferentially found at the 3′-end of transcriptionally active genes and does not co-localize with heterochromatic marks (Stroud et al., 2012b; Wollmann et al., 2012; Vaquero-Sedas and Vega-Palas, 2013). Therefore, both histones H3 variants target different genomic loci. Arabidopsis histones H3.1 and H3.3 differ in four residues. Residues at position 31 in both variants condition their association with heterochromatin or euchromatin. At this position, histones H3.1 and H3.3 contain alanine and threonine, respectively (Ray-Gallet and Almouzni, 2021). Whereas alanine in histone H3.1 allows monomethylation of lysine 27 by the ATXR5 and ATXR6 methyltransferases, threonine 31 in histone H3.3 impairs lysine 27 monomethylation. Thus, whereas histone H3.1 can be monomethylated at lysine 27, histone H3.3 should not be labeled with this heterochromatic mark (Jacob et al., 2009, 2010, 2014).

Telomeric regions, which include telomeres and subtelomeres, have been traditionally considered heterochromatic. However, whereas the heterochromatic nature of subtelomeres has been widely accepted, the epigenetic status of telomeres remains controversial. This has been largely due to the difficulty to study telomere epigenetics, not only in Arabidopsis but in many other organisms (Vaquero-Sedas and Vega-Palas, 2011). The epigenetic modifications of telomeres are usually analyzed by microscopy or by chromatin immunoprecipitation (ChIP). However, these analyses could be challenged by subtelomeres and/or ITSs. Whereas telomeres and subtelomeres cannot be differentiated by standard microscopy techniques, telomeres and ITSs might not be differentiated in ChIP analyses because both kinds of loci contain telomeric repeats. In addition, ChIP analyses of telomeres should be properly controlled. Hence, studies focused on the epigenetic features of telomeres have to be carefully designed and interpreted (Vaquero-Sedas and Vega-Palas, 2019).

Here, we have studied the epigenetic status of Arabidopsis telomeres by analyzing multiple genome-wide ChIP-seq experiments. Since we have been able to differentiate between telomeric reads and reads arising from ITSs, these ChIP-seq analyses have allowed us to study the epigenetic features of telomeres independently of ITSs with high levels of statistical confidence (see “Materials and methods”). This revealed that, in relation to the whole genome, Arabidopsis telomeres are not enriched in H3K4me2, H3K9ac, H3K27me3, H3K27me1, or H3K9me2. Our results support that telomeric regions in Arabidopsis have a bimodal chromatin organization with telomeres lacking significant levels of canonical euchromatic and heterochromatic modifications and subtelomeres organized as heterochromatin.

## Results

### Preferential association of epigenetic marks with single copy and repetitive DNA

**Figure 1:**
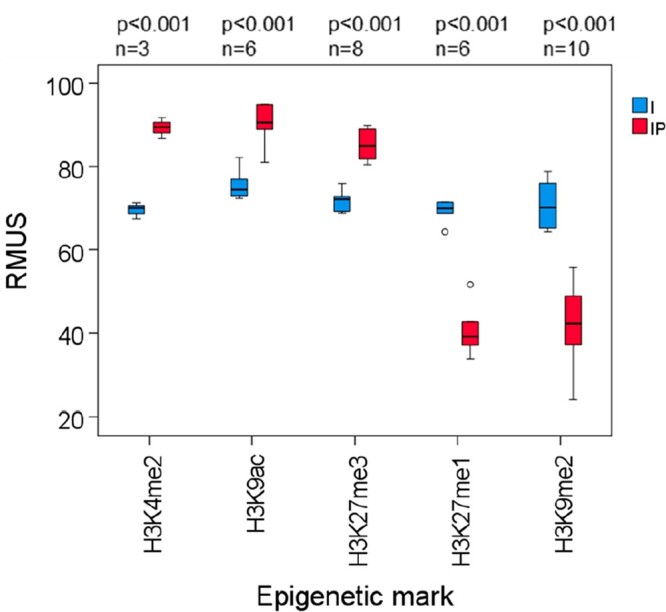
Euchromatic and heterochromatic marks preferentially associate with single-copy and repetitive DNA sequences, respectively. Whereas euchromatic marks (H3K4me2, H3K9ac, and H3K27me3) are shown in the left, heterochromatic marks (H3K27me1 and H3K9me2) are shown in the right. Box plots represent the percentages of input (I) and immunoprecipitated (IP) reads mapping to unique sequences (RMUS) for the different epigenetic modifications. Elements in each boxplot: center line, median; box limits, upper and lower quartiles; whiskers, 1.5× interquartile range; and points, outliers. The number of experiments analyzed (*n*) and the statistical levels of enrichment or depletion significance (*p*) are also indicated. *P* values were determined using the Student’s *t* test (H3K4me2, H3K9ac, H3K27me1, and H3K9me2) or the test of Kolmogorov–Smirnov (H3K27me3), depending on whether the distributions of enrichments were normal or not according to the Shapiro–Wilk test. This figure was made using the data provided in Supplemental Table S1.

Since heterochromatin is known to be enriched in different kinds of repetitive elements and euchromatin usually associates with gene-rich regions, we decided to analyze whether some Arabidopsis epigenetic marks that label euchromatin or heterochromatin associate preferentially with single copy or repetitive DNA sequences. We focused on three euchromatic marks (H3K4me2, H3K9ac, and H3K27me3) and on two heterochromatic marks (H3K27me1 and H3K9me2). Not surprisingly, we found that whereas the euchromatic marks associate preferentially with single copy sequences of the Arabidopsis genome, the heterochromatic marks tend to associate with repetitive sequences (Figure 1). This result corroborates that these epigenetic marks are hallmarks of euchromatin and heterochromatin, respectively, and, therefore, faithfully allows the analysis of the epigenetic status of Arabidopsis telomeres.

### Arabidopsis telomeres are not enriched in canonical euchromatic or heterochromatic marks

**Figure 2:**
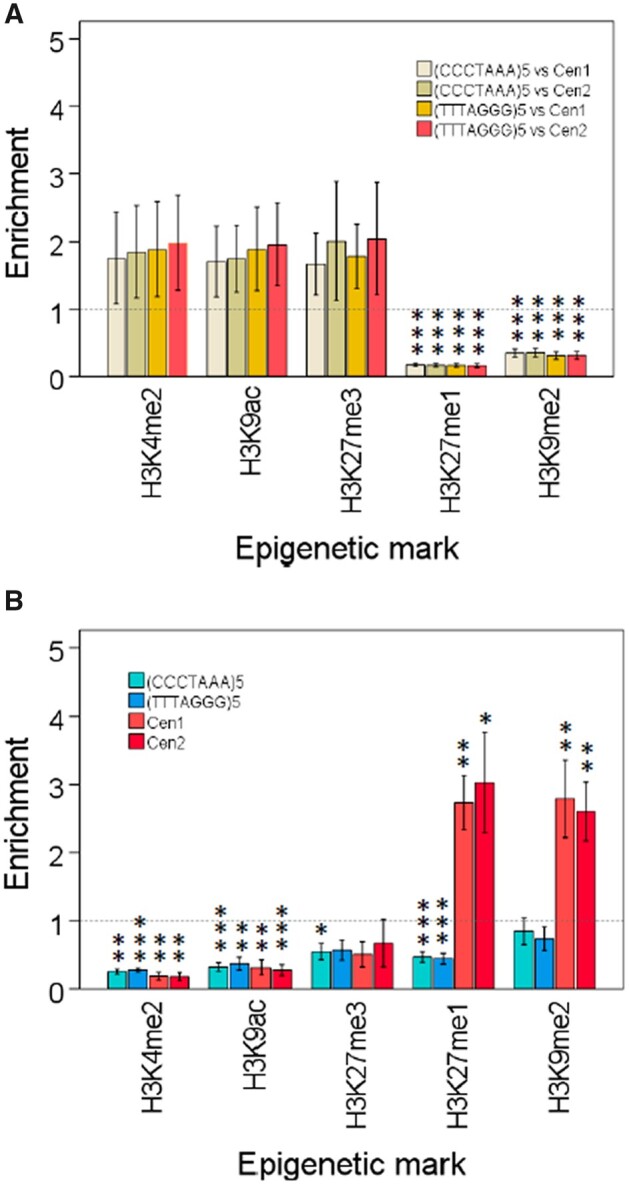
Arabidopsis telomeres are not enriched in canonical euchromatic or heterochromatic marks. A, Enrichment levels of the different euchromatic and heterochromatic marks at telomeres [(CCCTAAA)_5_ and (TTTAGGG)_5_] versus the 178 bp satellite repeats (Cen1 and Cen2). B, Enrichment levels of the different histone modifications at telomeres and at satellite repeats with regard to the whole genome. In both panels, sample sizes for the different epigenetic modifications are the same than in Figure 1, error bars represent the standard error of the mean, and asterisks label significantly enriched or depleted modifications. The levels of significance are as follows: *P < 0.05, **P < 0.01, ***P < 0.001. The statistical test used in each case is indicated in Supplemental Table S1, which also contains additional statistical information.

To get insight into the epigenetic status of Arabidopsis telomeres, we decided to determine the enrichment levels of the different epigenetic marks at telomeres with regard to the whole Arabidopsis genome and also with regard to the 178 bp satellite repeats, which have been usually studied as heterochromatic reference (Fransz et al., 1998; Lindroth et al., 2001, 2004; Johnson et al., 2002; Zhang et al., 2008; Vaquero-Sedas and Vega-Palas, 2013). For each epigenetic mark, we analyzed multiple genome-wide ChIP-seq experiments available at the Sequence Read Archive of NCBI. In this way, we strengthened the statistical levels of significance of the ChIP-seq analyses. We detected higher levels of euchromatic marks at Arabidopsis telomeres than at the 178 bp satellite repeats (Figure 2A). However, these enhanced levels of euchromatic marks are not statistically significant. Even more, the levels of euchromatic marks at telomeres are not enriched with regard to the whole genome, with some of them being significantly depleted (Figure 2B). In turn, the levels of the heterochromatic marks are significantly lower at telomeres than at the satellite repeats (Figure 2A) and are significantly enriched at the satellite repeats with regard to the whole genome (Figure 2B). In addition, these heterochromatic marks are not enriched (H3K9me2) or are significantly depleted (H3K27me1) at telomeres with regard to the whole genome (Figure 2B). Thus, our ChIP-seq analyses show that Arabidopsis telomeres are not enriched in canonical euchromatic or heterochromatic marks with regard to the whole genome. By contrast, the 178 bp satellite repeats are enriched in heterochromatic marks.

The absence of heterochromatic marks enrichment at Arabidopsis telomeres might seem surprising considering its repetitiveness and that pericentromeric and subtelomeric ITSs are heterochromatic (Vaquero-Sedas et al., 2011; Farrell et al., 2022). However, the unique characteristics of telomeric sequences and their nucleosomal organization should contribute to explain why telomeres are not labeled with heterochromatic marks (see below).

## Discussion

Although the results shown here are compatible with our previous epigenetic analyses of Arabidopsis telomeres, they render a more complete and statistically significant view that should be highlighted. Following, we summarize our previous results and discuss them together with our current findings. In addition, we comment on additional epigenetic analyses of Arabidopsis telomeres.

Following an approach similar to the one reported here, we have previously studied the epigenetic features of Arabidopsis telomeres by analyzing a specific genome-wide ChIP-seq experiment (Vaquero-Sedas et al., 2012). In agreement with the results shown here, we found higher levels of euchromatic marks and lower levels of heterochromatic marks at Arabidopsis telomeres than at the 178 bp repeats. Whereas we detected higher levels of H3K4me2 and H3K9ac (250% and 300%, respectively), we found lower levels of H3K9me2 and H3K27me1 (about 10%) (Vaquero-Sedas et al., 2012; Luo et al., 2013). We observed similar results after analyzing a rice (*Oryza sativa*) ChIP-seq experiment. In rice, we detected higher levels of H3K4me2 and H3K9ac (190% and 270%, respectively) and lower levels of cytosine methylation (10%) at telomeres than at the heterochromatic Cent0 satellite repeats (Cheng et al., 2002; Lee et al., 2006; Yan et al., 2010; Vaquero-Sedas et al., 2012). In addition, we found that both Arabidopsis and rice telomeres had higher levels of H3K27me3 than the satellite repeats (about 400%). Thus, our previous ChIP-seq analyses revealed higher levels of euchromatic marks at telomeres than at heterochromatic satellite repeats. Based on these results, we argued that Arabidopsis telomeres are labeled with euchromatic marks and have low levels of heterochromatic marks. However, we could not calculate statistical levels of significance and did not address the enrichment of the different marks at telomeres with regard to the whole genome because the input sample was not available in one of the two ChIP-seq studies analyzed.

We have also analyzed previously the epigenetic features of Arabidopsis telomeres by ChIP followed by hybridization with a telomeric probe (ChIP-hyb) (Vaquero-Sedas et al., 2011). To accomplish this, we first had to set up the ChIP-hyb technique because 70% of the signal detected after hybridizing Arabidopsis genomic DNA with a telomeric probe under stringent conditions corresponds to ITSs (Gámez-Arjona et al., 2010). The different DNA sequence organizations of Arabidopsis telomeres and ITSs facilitated this task. In Arabidopsis, telomeres are essentially composed of perfect telomeric repeat arrays, which do not contain restriction sites. By contrast, ITSs usually contain short stretches of perfect telomeric repeats interspersed with degenerate telomeric repeats that contain restriction sites. By digesting the input and immunoprecipitated DNA samples obtained after performing ChIP experiments with a specific restriction endonuclease (Tru9I), we could separate telomeres from ITSs by electrophoresis (Vaquero-Sedas et al., 2011). Then, by comparing the hybridization signals obtained before and after digesting the input and immunoprecipitated DNA samples with Tru9I, we could estimate the enrichment levels of different epigenetic modifications at telomeres versus ITSs. We found higher levels of euchromatic marks such as H3K4me2 and H3K9ac at telomeres than at ITSs (about 170%). In addition, we detected lower levels of heterochromatic marks such as DNA methylation, H3K9me2, and H3K27me1 at telomeres than at ITSs (30%–50%) (Vaquero-Sedas et al., 2011). These results also prompted us to argue that Arabidopsis telomeres are labeled with euchromatic marks and have low levels of heterochromatic marks.

With regard to DNA methylation, we have also addressed the DNA methylation status of Arabidopsis telomeres by analyzing Whole Genome Bisulfite Sequencing (WGBS) experiments (Vega-Vaquero et al., 2016). WGBS experiments involve the treatment of DNA with sodium bisulfite, the PCR amplification of the resulting DNA samples, and the sequencing of the bisulfite modified DNA strand. Since bisulfite deaminates unmethylated cytosines generating uracil, unmethylated cytosines are detected as thymines after PCR amplification. By contrast, methylated cytosines are not modified by bisulfite and remain as cytosines after amplification (Frommer et al., 1992; Clark et al., 1994). Our analysis of WGBS studies showed that Arabidopsis telomeric cytosines are converted to thymines after bisulfite treatment. Therefore, they are not methylated. We further confirmed this result by performing methylation-dependent restriction enzyme analyses (Vega-Vaquero et al., 2016). Whereas the 178 bp repeats are readily digested with the methylation-dependent enzymes FspEI and MrcBC, telomeres are not digested by these enzymes. Thus, three different approaches including ChIP-hyb, the analysis of WGBS data, and restriction enzyme analyses support that Arabidopsis telomeres do not undergo cytosine methylation.

We have also studied previously the association of histones H3.1 and H3.3 with Arabidopsis telomeres by analyzing ChIP-seq experiments (Wong et al., 2009; Goldberg et al., 2010; Lewis et al., 2010; Vaquero-Sedas and Vega-Palas, 2013). In this case, we could determine telomeric enrichment values with regard to the whole genome. By analyzing two independent genome-wide ChIP-seq studies, we found that the levels of histone H3.3 are enriched at Arabidopsis telomeres with regard to the whole genome (about 450%) and with regard to the 178 bp satellite repeats (about 1400%). In turn, the levels of H3.1 are depleted at telomeres with regard to the whole genome and with regard to the satellite repeats (about 40%). Thus, we concluded that Arabidopsis telomeric DNA associates with nucleosomes that contain the histone H3.3 variant, which has also been found in mammal telomeres (Wong et al., 2009; Goldberg et al., 2010; Lewis et al., 2010; Vaquero-Sedas and Vega-Palas, 2013). In turn, Arabidopsis pericentromeric nucleosomes associate with histone H3.1 (Stroud et al., 2012b; Wollmann et al., 2012; Vaquero-Sedas and Vega-Palas, 2013).

Both our previous results and the results reported here reveal that Arabidopsis telomeres have low levels of heterochromatic marks including DNA methylation, H3K9me2, and H3K27me1. In addition, both sets of results display higher levels of H3K4me2, H3K9ac, and H3K27me3 at telomeres than at heterochromatic elements such as ITSs or 178 bp repeats. However, our current results reveal that these enhanced levels of euchromatic marks are not statistically significant. Even more, these euchromatic marks are not enriched at telomeres with regard to the whole genome. Therefore, telomeres are not significantly enriched in H3K4me2, H3K9ac, and H3K27me3.

Although the enhanced levels of euchromatic marks that we have detected at telomeres with regard to heterochromatic elements are not statistically significant, their validity is likely since we have detected them by different means. They could be explained by assuming that low levels of euchromatic marks associate with Arabidopsis telomeres and not with large heterochromatic blocks containing ITSs and 178 bp repeats. Alternatively, enhanced levels of euchromatic marks might be detected at telomeres due a border effect. This border effect entails the influence of subtelomeric chromatin on ChIP-hyb or ChIP-seq analyses of telomeres, as previously discussed (Vaquero-Sedas and Vega-Palas, 2019). Its magnitude should depend on the length of telomeres and of the immunoprecipitated chromatin fragments. Since subtelomeric heterochromatin only extends 1–2 kb from the telomere–subtelomere boundaries, antibodies against euchromatic marks located near to the centromeric side of subtelomeric heterochromatin might immunoprecipitate with low frequency DNA fragments containing the centromeric side of certain telomeres. In this context, it is interesting to note that there are transcriptionally active genes and H3K27me3 domains near to certain Arabidopsis telomere–subtelomere boundaries (see browser at https://gbrowse.mpipz.mpg.de/cgi-bin/gbrowse/arabidopsis10_turck_public for H3K27me3 domains) (Vrbsky et al., 2010; Dong et al., 2012; Zhou et al., 2018).

A border effect could also explain why H3K9me2 is not significantly depleted at telomeres with regard to the whole genome (Figure 2B). Since subtelomeric heterochromatin is enriched in H3K9me2, antibodies recognizing H3K9me2 might immunoprecipitate subtelomeric DNA fragments containing the centromeric side of telomeres with low frequency. Even more, these antibodies could directly immunoprecipitate the centromeric side of telomeres within the telomere–subtelomere boundaries, which undergo very low levels of cytosine methylation (Farrell et al., 2022). This border effect would not be observed for H3K27me1 because the levels of this epigenetic mark within subtelomeric heterochromatin are lower than those of H3K9me2 (M.I. Vaquero-Sedas and M.A. Vega-Palas, unpublished data).

Finally, a border effect could also help explain why some studies performed by other groups have reported the presence of euchromatic marks (H3K4me2, H3K4me3, and H3K27me3) and heterochromatic marks (cytosine methylation, H3K27me1, and H3K9me2) at telomeres after performing ChIP-hyb analyses (Vrbsky et al., 2010; Ogrocká et al., 2014; Sováková et al., 2018; Adamusová et al., 2020). However, ITSs might have also influenced those studies because the dot-blots hybridized after performing the ChIP experiments included both telomeres and ITSs.

## Concluding remarks

Telomeres in Arabidopsis have low levels of heterochromatic marks such as DNA methylation, H3K9me2, and H3K27me1. The low levels of H3K27me1 at Arabidopsis telomeres are in agreement with their association with the euchromatic histone H3.3 variant. Since histone H3.3 cannot be monomethylated at lysine 27 by ATXR5 and ATXR6, telomeric nucleosomes are not expected to be labeled with H3K27me1 (Vaquero-Sedas and Vega-Palas, 2013; Jacob et al., 2014). In addition, since Arabidopsis telomeres do not undergo DNA methylation, they are not expected to be labeled with H3K9me2 because DNA methylation is largely required for H3K9me2 and vice versa (Du et al., 2015; Vega-Vaquero et al., 2016). Thus, heterochromatic marks should be essentially absent from Arabidopsis telomeres. In addition, euchromatic marks like H3K4me2, H3K9ac, and H3K27me3 are not enriched at telomeres with regard to the whole genome. Therefore, Arabidopsis telomeres are not enriched in canonical euchromatic or heterochromatic marks.

The nature of telomeric chromatin is certainly singular because it has short nucleosomes containing histone H3.3 and associates with telomeric proteins involved in telomere protection and replication (Blackburn et al., 2015). Hence, it doesn’t fit well within the canonical classification of chromatin into euchromatin or heterochromatin. However, the proper function of Arabidopsis or human telomeres requires the integrity of heterochromatin (Nishibuchi and Déjardin, 2017; de Lange, 2018). Indeed, mutations in DNA or histone methyltransferases involved in heterochromatin maintenance are known to shorten Arabidopsis telomeres (Vaquero-Sedas et al., 2011; Ogrocká et al., 2014; Vaquero-Sedas and Vega-Palas, 2014). Considering that subtelomeres are heterochromatic and that heterochromatin influences telomere function, we decided to refer to the chromatin organization of Arabidopsis telomeric regions as bimodal. This bimodal organization includes telomeres lacking significant levels of canonical euchromatic and heterochromatic marks and subtelomeres labeled with heterochromatic modifications that could play a relevant role in telomere biology (Vaquero-Sedas et al., 2011; Vega-Vaquero et al., 2016).

## Materials and methods

### Identification of telomeric and 178 bp repeats reads in ChIP-seq studies

We have identified the DNA sequences that reveal telomeres but not ITSs in genome-wide ChIP-seq experiments when using the recently released Col-XJTU Arabidopsis (*A. thaliana*) genome as reference (Wang et al., 2021). This more recent version of the genome has been assembled using Nanopore and HiFi long reads and is more complete and accurate than TAIR10. It includes more repetitive DNA sequences and, therefore, contains more centromeric and pericentromeric repeats. We estimated the number of times that the sequence (CCCTAAA)_5_ appears at internal chromosomal loci and at Arabidopsis telomeres. For that purpose, we first determined the number of times that the sequence (CCCTAAA)_5_ appears at internal chromosomal loci in the Col-XJTU genome. In the case that a specific ITS contained six perfect tandem telomeric repeats, we counted two overlapping (CCCTAAA)_5_ sequences. If the ITS contained seven perfect tandem telomeric repeats, we counted three overlapping (CCCTAAA)_5_ sequences and so on. We found 109 (CCCTAAA)_5_ sequences at internal positions in the five chromosomes of the Arabidopsis Col-XJTU genome, including subtelomeric regions. To estimate the number of times that the sequence (CCCTAAA)_5_ is found at Arabidopsis telomeres we didn’t use the Col-XJTU genome because it does not contain the complete sequences of all telomeres. Instead, we assumed that *A. thaliana* (Col-0) telomeres are composed of perfect tandem telomeric repeats arrays that spread about 3,750 bp, which is supported by previous reports (Richards and Ausubel, 1988; Shakirov and Shippen, 2004). This notion is also supported by the Col-XJTU genome, which mainly contains perfect arrays of tandem telomeric repeats at telomeres. We estimated that the five Arabidopsis chromosomes should contain about 5,350 overlapping (CCCTAAA)_5_ sequences at telomeres [(3750/7) × 10]. Thus, when the frequency of reads containing the sequence (CCCTAAA)_5_ is determined in input samples of Arabidopsis ChIP-seq experiments, only 2% of these reads should correspond to ITSs [(109 × 100)/(109 + 5350)]. Consequently, perfect arrays of telomeric repeats containing five repeats or more represent telomeres in Arabidopsis ChIP-seq experiments. Therefore, we decided to identify as telomeric reads those containing the sequences (CCCTAAA)_5_ or (TTTAGGG)_5_. Most of these reads only contain tandem arrays of the corresponding telomeric repeats. We counted both kinds of telomeric reads in the input and immunoprecipitated DNA samples and used them to calculate telomeric enrichment values. In addition, and for comparison, reads containing the sequences TTGGCTTTGTATCTTCTAACAAG (Cen1) and CATATTTGACTCCAAAACACTAA (Cen2) were counted and used to calculate the enrichment levels at the 178 bp satellite repeats, which served as heterochromatic reference (Zhang et al., 2008). Although a fraction of the 178 bp sequences associates with CENH3 chromatin, the surrounding 178 bp repeats associate with H3.1 chromatin. Since Cen1 and Cen2 do not contain motifs specifically associated with CENH3 chromatin (Zhang et al., 2008), these sequences are present at the 178 bp repeats that associate with CENH3 chromatin and also at the 178 bp repeats that associate with H3.1 chromatin. Thus, they allowed us to analyze the chromatin of the 178 bp satellite repeats as an average, which is known to be enriched in heterochromatic marks (Fransz et al., 1998; Lindroth et al., 2001, 2004; Johnson et al., 2002; Zhang et al., 2008; Vaquero-Sedas and Vega-Palas, 2013). Therefore, four different kinds of reads arising from telomeres or from the 178 bp repeats were analyzed in this study.

### Determination of enrichment levels

First, for every ChIP-seq study analyzed, the input and immunoprecipitated Fastq files were uploaded from the NCBI Sequence Read Archive to Galaxy public servers (https://usegalaxy.org and https://usegalaxy.eu) and aligned with the Arabidopsis Col-XJTU reference genome using Bowtie2 with default parameters (Afgan et al., 2016; Galaxy Community, 2022). The mapping reads were selected and counted, and the number of telomeric and 178 bp reads were determined by using the filter and sort and the text manipulation options at the servers. Then, the frequencies of telomeric and 178 bp repeats reads in the input and immunoprecipitated samples were calculated by dividing the number of each kind of read between the total number of mapping reads.

For each study, enrichment values at telomeres or at the 178 bp repeats with regard to the whole genome were calculated by dividing the immunoprecipitation frequencies between the frequencies of the corresponding input samples. In addition, enrichment values at telomeres versus the 178 bp satellite repeats were calculated by dividing the telomeric reads enrichment values between the 178 bp reads values. Then, for every specific mark, enrichment values from different studies were pooled together and statistical levels of significance were determined using the Student’s *t* test or the test of Wilcoxon, depending on whether the distributions of enrichments were normal or not according to the Shapiro–Wilk test (Supplemental Table S1).

### Determination of the percentages of reads mapping to unique sequences

For every input and immunoprecipitated sample analyzed, reads mapping to unique sequences (RMUS) values were calculated by dividing the number of reads that map only once to the Arabidopsis genome between the total number of mapping reads. Both kinds of data were obtained after aligning the Fastq files to the Arabidopsis Col-XJTU reference genome with Bowtie2. Then, for every epigenetic mark, RMUS corresponding to the input samples of different studies were pooled together as well as RMUS corresponding to immunoprecipitated samples. Statistical levels of significance of pair-wise comparisons were performed as mentioned above (Supplemental Table S1).

As previously mentioned, the results shown in this manuscript have been elaborated using the Col-XJTU genome that has been recently released, which does not include the chloroplast and mitochondrial genomes (Wang et al., 2021). Interestingly, similar results are obtained when using the Col-XJTU genome plus the chloroplast and mitochondrial genomes (NCBI Reference Sequences NC_000932.1 and NC_037304.1, respectively) or the build in TAIR10 reference genome displayed in the Galaxy servers, which also include the genomes of the organelles (see Supplemental Table S2).

## Supplemental data

The following materials are available in the online version of this article.

**Supplemental Table S1.** Determination of enrichment values and statistical levels of significance.

**Supplemental Table S2.** Summary of enrichment data using different reference genomes for alignment.

## Supplementary Material

kiac471_Supplementary_Data

## Notes

M.A.V.-P. and M.I.V.-S. performed the analyses and wrote the manuscript.

The author responsible for distribution of materials integral to the findings presented in this article in accordance with the policy described in the Instructions for Authors (https://academic.oup.com/plphys/pages/general-instructions) is: Miguel Á. Vega-Palas (vega-palas@ibvf.csic.es).

## References

[kiac471-B1] Adamusová K , Khosravi S, Fujimoto S, Houben A, Matsunaga S, Fajkus J, Fojtová M (2020) Two combinatorial patterns of telomere histone marks in plants with canonical and non-canonical telomere repeats. Plant J 102: 678–687 31834959 10.1111/tpj.14653

[kiac471-B2] Afgan E , Baker D, van den Beek M, Blankenberg D, Bouvier D, Čech M, Chilton J, Clements D, Coraor N, Eberhard C, et al (2016) The Galaxy platform for accessible, reproducible and collaborative biomedical analyses: 2016 update. Nucleic Acids Res 44: W3–W10 27137889 10.1093/nar/gkw343 PMC4987906

[kiac471-B3] Aksenova AY , Mirkin SM (2019) At the beginning of the end and in the middle of the beginning: structure and maintenance of telomeric DNA repeats and interstitial telomeric sequences. Genes (Basel) 10: 118 30764567 10.3390/genes10020118 PMC6410037

[kiac471-B4] Blackburn EH , Epel ES, Lin J (2015) Human telomere biology: a contributory and interactive factor in aging, disease risks, and protection. Science 350: 1193–1198 26785477 10.1126/science.aab3389

[kiac471-B5] Cheng Z , Dong F, Langdon T, Ouyang S, Buell CR, Gu M, Blattner FR, Jiang J (2002) Functional rice centromeres are marked by a satellite repeat and a centromere-specific retrotransposon. Plant Cell 14: 1691–1704 12172016 10.1105/tpc.003079 PMC151459

[kiac471-B6] Clark SJ , Harrison J, Paul CL, Frommer M (1994) High sensitivity mapping of methylated cytosines. Nucleic Acids Res 22: 2990–2997 8065911 10.1093/nar/22.15.2990 PMC310266

[kiac471-B7] Dong X , Reimer J, Göbel U, Engelhorn J, He F, Schoof H, Turck F (2012) Natural variation of H3K27me3 distribution between two Arabidopsis accessions and its association with flanking transposable elements. Genome Biol 13: R117 23253144 10.1186/gb-2012-13-12-r117 PMC4056368

[kiac471-B8] Du J , Johnson LM, Jacobsen SE, Patel DJ (2015) DNA methylation pathways and their crosstalk with histone methylation. Nat Rev Mol Cell Biol 16: 519–532 26296162 10.1038/nrm4043 PMC4672940

[kiac471-B9] Du J , Zhong X, Bernatavichute YV, Stroud H, Feng S, Caro E, Vashisht AA, Terragni J, Chin HG, Tu A, et al (2012) Dual binding of chromomethylase domains to H3K9me2-containing nucleosomes directs DNA methylation in plants. Cell 151: 167–180 23021223 10.1016/j.cell.2012.07.034 PMC3471781

[kiac471-B10] Ebbs ML , Bender J (2006) Locus-specific control of DNA methylation by the Arabidopsis SUVH5 histone methyltransferase. Plant Cell 18: 1166–1176 16582009 10.1105/tpc.106.041400 PMC1456864

[kiac471-B11] Farrell C , Vaquero-Sedas MI, Cubiles MD, Thompson M, Vega-Vaquero A, Pellegrini M, Vega-Palas MA (2022) A complex network of interactions governs DNA methylation at telomeric regions. Nucleic Acids Res 50: 1449–1464 35061900 10.1093/nar/gkac012 PMC8860613

[kiac471-B12] Feng W , Hale CJ, Over RS, Cokus SJ, Jacobsen SE, Michaels SD (2017) Large-scale heterochromatin remodeling linked to overreplication-associated DNA damage. Proc Natl Acad Sci USA 114: 406–411 28028228 10.1073/pnas.1619774114 PMC5240675

[kiac471-B13] Fransz P , Armstrong S, Alonso-Blanco C, Fischer TC, Torres-Ruiz RA, Jones G (1998) Cytogenetics for the model system *Arabidopsis thaliana*. Plant J 13: 867–876 9681023 10.1046/j.1365-313x.1998.00086.x

[kiac471-B14] Frommer M , McDonald LE, Millar DS, Collis CM, Watt F, Grigg GW, Molloy PL, Paul CL (1992) A genomic sequencing protocol that yields a positive display of 5-methylcytosine residues in individual DNA strands. Proc Natl Acad Sci USA 89: 1827–1831 1542678 10.1073/pnas.89.5.1827 PMC48546

[kiac471-B15] Fuchs J , Demidov D, Houben A, Schubert I (2006) Chromosomal histone modification patterns—from conservation to diversity. Trends Plant Sci 11: 199–208 16546438 10.1016/j.tplants.2006.02.008

[kiac471-B16] Galaxy Community (2022) The Galaxy platform for accessible, reproducible and collaborative biomedical analyses: 2022 update. Nucleic Acids Resgkac247 35446428 10.1093/nar/gkac247 PMC9252830

[kiac471-B17] Gámez-Arjona FM , López-López C, Vaquero-Sedas MI, Vega-Palas MA (2010) On the organization of the nucleosomes associated with telomeric sequences. Biochim Biophys Acta 1803: 1058–1061 20381544 10.1016/j.bbamcr.2010.03.021

[kiac471-B18] Goldberg AD , Banaszynski LA, Noh K-M, Lewis PW, Elsaesser SJ, Stadler S, Dewell S, Law M, Guo X, Li X, et al (2010) Distinct factors control histone variant H3.3 localization at specific genomic regions. Cell 140: 678–691 20211137 10.1016/j.cell.2010.01.003 PMC2885838

[kiac471-B19] Jackson JP , Johnson L, Jasencakova Z, Zhang X, Perez-Burgos L, Singh PB, Cheng X, Schubert I, Jenuwein T, Jacobsen SE (2004) Dimethylation of histone H3 lysine 9 is a critical mark for DNA methylation and gene silencing in *Arabidopsis thaliana*. Chromosoma 112: 308–315 15014946 10.1007/s00412-004-0275-7

[kiac471-B20] Jackson JP , Lindroth AM, Cao X, Jacobsen SE (2002) Control of CpNpG DNA methylation by the KRYPTONITE histone H3 methyltransferase. Nature 416: 556–560 11898023 10.1038/nature731

[kiac471-B21] Jacob Y , Bergamin E, Donoghue MTA, Mongeon V, LeBlanc C, Voigt P, Underwood CJ, Brunzelle JS, Michaels SD, Reinberg D, et al (2014) Selective methylation of histone H3 variant H3.1 regulates heterochromatin replication. Science 343: 1249–1253 24626927 10.1126/science.1248357 PMC4049228

[kiac471-B22] Jacob Y , Feng S, LeBlanc CA, Bernatavichute YV, Stroud H, Cokus S, Johnson LM, Pellegrini M, Jacobsen SE, Michaels SD (2009) ATXR5 and ATXR6 are H3K27 monomethyltransferases required for chromatin structure and gene silencing. Nat Struct Mol Biol 16: 763–768 19503079 10.1038/nsmb.1611 PMC2754316

[kiac471-B23] Jacob Y , Stroud H, Leblanc C, Feng S, Zhuo L, Caro E, Hassel C, Gutierrez C, Michaels SD, Jacobsen SE (2010) Regulation of heterochromatic DNA replication by histone H3 lysine 27 methyltransferases. Nature 466: 987–991 20631708 10.1038/nature09290 PMC2964344

[kiac471-B24] Johnson L , Cao X, Jacobsen S (2002) Interplay between two epigenetic marks. DNA methylation and histone H3 lysine 9 methylation. Curr Biol 12: 1360–1367 12194816 10.1016/s0960-9822(02)00976-4

[kiac471-B25] Jung N , Kim T-K (2021) Advances in higher-order chromatin architecture: the move towards 4D genome. BMB Rep 54: 233–245 33972012 10.5483/BMBRep.2021.54.5.035 PMC8167246

[kiac471-B26] de Lange T (2018) Shelterin-mediated telomere protection. Annu Rev Genet 52: 223–247 30208292 10.1146/annurev-genet-032918-021921

[kiac471-B27] Law JA , Du J, Hale CJ, Feng S, Krajewski K, Palanca AMS, Strahl BD, Patel DJ, Jacobsen SE (2013) Polymerase IV occupancy at RNA-directed DNA methylation sites requires SHH1. Nature 498: 385–389 23636332 10.1038/nature12178 PMC4119789

[kiac471-B28] Law JA , Vashisht AA, Wohlschlegel JA, Jacobsen SE (2011) SHH1, a homeodomain protein required for DNA methylation, as well as RDR2, RDM4, and chromatin remodeling factors, associate with RNA polymerase IV. PLoS Genet 7: e1002195 21811420 10.1371/journal.pgen.1002195 PMC3141008

[kiac471-B29] Lee H-R , Neumann P, Macas J, Jiang J (2006) Transcription and evolutionary dynamics of the centromeric satellite repeat CentO in rice. Mol Biol Evol 23: 2505–2520 16987952 10.1093/molbev/msl127

[kiac471-B30] Lewis PW , Elsaesser SJ, Noh K-M, Stadler SC, Allis CD (2010) Daxx is an H3.3-specific histone chaperone and cooperates with ATRX in replication-independent chromatin assembly at telomeres. Proc Natl Acad Sci USA 107: 14075–14080 20651253 10.1073/pnas.1008850107 PMC2922592

[kiac471-B31] Lindroth AM , Cao X, Jackson JP, Zilberman D, McCallum CM, Henikoff S, Jacobsen SE (2001) Requirement of CHROMOMETHYLASE3 for maintenance of CpXpG methylation. Science 292: 2077–2080 11349138 10.1126/science.1059745

[kiac471-B32] Lindroth AM , Shultis D, Jasencakova Z, Fuchs J, Johnson L, Schubert D, Patnaik D, Pradhan S, Goodrich J, Schubert I, et al (2004) Dual histone H3 methylation marks at lysines 9 and 27 required for interaction with CHROMOMETHYLASE3. EMBO J 23: 4286–4296 15457214 10.1038/sj.emboj.7600430 PMC524394

[kiac471-B33] Luger K , Mäder AW, Richmond RK, Sargent DF, Richmond TJ (1997) Crystal structure of the nucleosome core particle at 2.8 A resolution. Nature 389: 251–260 9305837 10.1038/38444

[kiac471-B34] Luo C , Sidote DJ, Zhang Y, Kerstetter RA, Michael TP, Lam E (2013) Integrative analysis of chromatin states in Arabidopsis identified potential regulatory mechanisms for natural antisense transcript production. Plant J 73: 77–90 22962860 10.1111/tpj.12017

[kiac471-B35] Mathieu O , Probst AV, Paszkowski J (2005) Distinct regulation of histone H3 methylation at lysines 27 and 9 by CpG methylation in Arabidopsis. EMBO J 24: 2783–2791 16001083 10.1038/sj.emboj.7600743 PMC1182238

[kiac471-B36] Nishibuchi G , Déjardin J (2017) The molecular basis of the organization of repetitive DNA-containing constitutive heterochromatin in mammals. Chromosome Res 25: 77–87 28078514 10.1007/s10577-016-9547-3

[kiac471-B37] Ogrocká A , Polanská P, Majerová E, Janeba Z, Fajkus J, Fojtová M (2014) Compromised telomere maintenance in hypomethylated *Arabidopsis thaliana* plants. Nucleic Acids Res 42: 2919–2931 24334955 10.1093/nar/gkt1285 PMC3950684

[kiac471-B38] Ray-Gallet D , Almouzni G (2021) The histone H3 family and its deposition pathways. Adv Exp Med Biol 1283: 17–42 33155135 10.1007/978-981-15-8104-5_2

[kiac471-B39] Richards EJ , Ausubel FM (1988) Isolation of a higher eukaryotic telomere from *Arabidopsis thaliana*. Cell 53: 127–136 3349525 10.1016/0092-8674(88)90494-1

[kiac471-B40] Roudier F , Ahmed I, Bérard C, Sarazin A, Mary-Huard T, Cortijo S, Bouyer D, Caillieux E, Duvernois-Berthet E, Al-Shikhley L, et al (2011) Integrative epigenomic mapping defines four main chromatin states in Arabidopsis. EMBO J 30: 1928–1938 21487388 10.1038/emboj.2011.103 PMC3098477

[kiac471-B41] Schuettengruber B , Bourbon H-M, Di Croce L, Cavalli G (2017) Genome regulation by Polycomb and Trithorax: 70 years and counting. Cell 171: 34–57 28938122 10.1016/j.cell.2017.08.002

[kiac471-B42] Shakirov EV , Shippen DE (2004) Length regulation and dynamics of individual telomere tracts in wild-type Arabidopsis. Plant Cell 16: 1959–1967 15258263 10.1105/tpc.104.023093 PMC519188

[kiac471-B43] Sováková PP , Magdolenová A, Konečná K, Rájecká V, Fajkus J, Fojtová M (2018) Telomere elongation upon transfer to callus culture reflects the reprogramming of telomere stability control in Arabidopsis. Plant Mol Biol 98: 81–99 30128721 10.1007/s11103-018-0765-2

[kiac471-B44] Stroud H , Do T, Du J, Zhong X, Feng S, Johnson L, Patel DJ, Jacobsen SE (2014) Non-CG methylation patterns shape the epigenetic landscape in Arabidopsis. Nat Struct Mol Biol 21: 64–72 24336224 10.1038/nsmb.2735 PMC4103798

[kiac471-B45] Stroud H , Greenberg MVC, Feng S, Bernatavichute YV, Jacobsen SE (2013) Comprehensive analysis of silencing mutants reveals complex regulation of the Arabidopsis methylome. Cell 152: 352–364 23313553 10.1016/j.cell.2012.10.054 PMC3597350

[kiac471-B46] Stroud H , Hale CJ, Feng S, Caro E, Jacob Y, Michaels SD, Jacobsen SE (2012a) DNA methyltransferases are required to induce heterochromatic re-replication in Arabidopsis. PLoS Genet 8: e1002808 22792077 10.1371/journal.pgen.1002808 PMC3390372

[kiac471-B47] Stroud H , Otero S, Desvoyes B, Ramírez-Parra E, Jacobsen SE, Gutierrez C (2012b) Genome-wide analysis of histone H3.1 and H3.3 variants in *Arabidopsis thaliana*. Proc Natl Acad Sci USA 109: 5370–5375 22431625 10.1073/pnas.1203145109 PMC3325649

[kiac471-B48] Vaquero-Sedas MI , Gámez-Arjona FM, Vega-Palas MA (2011) *Arabidopsis thaliana* telomeres exhibit euchromatic features. Nucleic Acids Res 39: 2007–2017 21071395 10.1093/nar/gkq1119 PMC3064777

[kiac471-B49] Vaquero-Sedas MI , Luo C, Vega-Palas MA (2012) Analysis of the epigenetic status of telomeres by using ChIP-seq data. Nucleic Acids Res 40: e163 22855559 10.1093/nar/gks730 PMC3505975

[kiac471-B50] Vaquero-Sedas MI , Vega-Palas MA (2013) Differential association of Arabidopsis telomeres and centromeres with histone H3 variants. Sci Rep 3: 1202 23383372 10.1038/srep01202 PMC3563029

[kiac471-B51] Vaquero-Sedas MI , Vega-Palas MA (2011) On the chromatin structure of eukaryotic telomeres. Epigenetics 6: 1055–1058 21822057 10.4161/epi.6.9.16845 PMC3225743

[kiac471-B52] Vaquero-Sedas MI , Vega-Palas MA (2019) Assessing the epigenetic status of human telomeres. Cells. doi:10.3390/cells8091050 PMC6770363 31500249

[kiac471-B53] Vaquero-Sedas MI , Vega-Palas MA (2014) Determination of *Arabidopsis thaliana* telomere length by PCR. Sci Rep 4: 5540 24986269 10.1038/srep05540 PMC4078305

[kiac471-B54] Vega-Vaquero A , Bonora G, Morselli M, Vaquero-Sedas MI, Rubbi L, Pellegrini M, Vega-Palas MA (2016) Novel features of telomere biology revealed by the absence of telomeric DNA methylation. Genome Res 26: 1047–1056 27405804 10.1101/gr.202465.115 PMC4971770

[kiac471-B55] Venditti S , Vega-Palas MA, Di Stefano G, Di Mauro E (1999) Imbalance in dosage of the genes for the heterochromatin components Sir3p and histone H4 results in changes in the length and sequence organization of yeast telomeres. Mol Gen Genet 262: 367–377 10517334 10.1007/s004380051095

[kiac471-B56] Vergara Z , Gutierrez C (2017) Emerging roles of chromatin in the maintenance of genome organization and function in plants. Genome Biol 18: 96 28535770 10.1186/s13059-017-1236-9 PMC5440935

[kiac471-B57] Vrbsky J , Akimcheva S, Watson JM, Turner TL, Daxinger L, Vyskot B, Aufsatz W, Riha K (2010) siRNA-mediated methylation of Arabidopsis telomeres. PLoS Genet 6: e1000986 20548962 10.1371/journal.pgen.1000986 PMC2883606

[kiac471-B58] Wang B , Yang X, Jia Y, Xu Y, Jia P, Dang N, Wang S, Xu T, Zhao X, Gao S, et al (2021) High-quality *Arabidopsis thaliana* genome assembly with nanopore and HiFi long reads. Genom Proteom Bioinform 20: 4–13 10.1016/j.gpb.2021.08.003 PMC9510872 34487862

[kiac471-B59] Wendte JM , Schmitz RJ (2018) Specifications of targeting heterochromatin modifications in plants. Mol Plant 11: 381–387 29032247 10.1016/j.molp.2017.10.002

[kiac471-B60] Wollmann H , Holec S, Alden K, Clarke ND, Jacques P-É, Berger F (2012) Dynamic deposition of histone variant H3.3 accompanies developmental remodeling of the Arabidopsis transcriptome. PLoS Genet 8: e1002658 22570629 10.1371/journal.pgen.1002658 PMC3342937

[kiac471-B61] Wong LH , Ren H, Williams E, McGhie J, Ahn S, Sim M, Tam A, Earle E, Anderson MA, Mann J, et al (2009) Histone H3.3 incorporation provides a unique and functionally essential telomeric chromatin in embryonic stem cells. Genome Res 19: 404–414 19196724 10.1101/gr.084947.108 PMC2661805

[kiac471-B62] Yan H , Kikuchi S, Neumann P, Zhang W, Wu Y, Chen F, Jiang J (2010) Genome-wide mapping of cytosine methylation revealed dynamic DNA methylation patterns associated with genes and centromeres in rice. Plant J 63: 353–365 20487381 10.1111/j.1365-313X.2010.04246.x

[kiac471-B63] Yin X , Romero-Campero FJ, de Los Reyes P, Yan P, Yang J, Tian G, Yang X, Mo X, Zhao S, Calonje M, et al (2021) H2AK121ub in Arabidopsis associates with a less accessible chromatin state at transcriptional regulation hotspots. Nat Commun 12: 315 33436613 10.1038/s41467-020-20614-1 PMC7804394

[kiac471-B64] Zhang W , Lee H-R, Koo D-H, Jiang J (2008) Epigenetic modification of centromeric chromatin: hypomethylation of DNA sequences in the CENH3-associated chromatin in *Arabidopsis thaliana* and maize. Plant Cell 20: 25–34 18239133 10.1105/tpc.107.057083 PMC2254920

[kiac471-B65] Zhou Y , Wang Y, Krause K, Yang T, Dongus JA, Zhang Y, Turck F (2018) Telobox motifs recruit CLF/SWN-PRC2 for H3K27me3 deposition via TRB factors in Arabidopsis. Nat Genet 50: 638–644 29700471 10.1038/s41588-018-0109-9

